# Stigma and Relationship Quality: The Relevance of Racial-Ethnic Worldview in Interracial Relationships in the United States

**DOI:** 10.3389/fpsyg.2022.923019

**Published:** 2022-07-12

**Authors:** James E. Brooks, Megan M. Morrison

**Affiliations:** ^1^Department of Counseling and Educational Psychology, Indiana University, Bloomington, IN, United States; ^2^Department of Psychology, Tennessee State University, Nashville, TN, United States

**Keywords:** interracial relationships, relationship quality, stigma, Racial-ethnic Worldview, race, discrimination

## Abstract

The current study explored the associations between relationship stigma, Racial-ethnic Worldview (REW; a construct developed as a comprehensive assessment of individual's perceptions of race and ethnicity), and relationship quality among those in interracial relationships (i. e., participants indicated their race was different than the race of their partner). One type of REW (Color-blind Achieved) was especially susceptible to the negative consequences of stigma from family members. Other significant differences in relationship quality and relationship stigma were found based on REW. Most notable is that individuals who acknowledge institutional racism, have positive intergroup attitudes, and a positive ethnic identity reported better relationship quality than those who denied institutional racism and/or had less positive attitudes toward their own ethnic group. These results demonstrate the utility of REW in contextualizing the experiences of individuals in interracial relationships as it relates to perceived stigma and relationship quality. The study offers a critical account of how individuals understanding of the racial and ethnic social context shapes relationship outcomes for those in interracial relationships in the United States.

## Introduction

Comparative studies are often used to gain a better understanding of the experiences of those in interracial relationships. These investigations often center, as normative, on the experiences, structure, and worldview of white and eurocentric ideals and conclusions often favor same-race relationships. In some ways, this approach has led to a pathologizing of interracial relationships. For example, investigations into higher divorce rates in interracial marriages explain disparities as a matter of “cultural differences” or as a consequence of public opposition because of facets such as racial composition (Brooks, 2022a). Conclusions often suggested poorer outcomes for interracial relationships (Fu et al., 2001; Hohmann-Marriott and Amato, 2008). Though some scholars explore interracial relationships by examining specific racial compositions of couples, they often rely on broad group-level differences and mask the heterogeneity within relationships and thus the nuance of race and ethnicity (Troy et al., 2006; Johns et al., 2007). As part of this special issue, we will ground the experiences of those in interracial relationships with attention to the race-based power structures that are characteristic of the United States, a western, educated, industrialized, rich, democratic (WEIRD) society. For the purposes of this study, interracial relationships are defined as intimate pairings of individuals in which a person's racial self-identification is different than their partner's (Brooks, 2022b). Included in this group are mono-racial individuals of different group identifications and relationships in which one or both partners identify as multiracial but there is a difference in racial group heritage between partners (e.g., a person of Black/White racial background in a relationship with a person of Latinx/White background).

Here we propose an alternative to the use of a comparison paradigm and instead focus on using a critical exploration of how the understanding of race impacts those in interracial relationships. We do so by calling attention to the Racial-ethnic Worldview, a comprehensive understanding of how individuals understand the impact of race in their lived experiences. In demonstrating the utility of the Racial-ethnic Worldview, we expand upon literature that addresses the lived experiences of discrimination of individuals in interracial relationships and the implications for relationship quality.

### Stigma and Discrimination in Interracial Relationships

Despite their increase in numbers (Livingston and Brown, 2017), those who partner across racial lines still experience a great deal of stigma and discrimination. Despite trends of expressed approval across the United States, many still would not date or marry someone of a different race (Herman and Campbell, 2012). Some perceive interracial relationships as of lesser quality than same-race relationships (Lewandowski and Jackson, 2001; Garcia et al., 2012). The perception of this marginalization impacts reports of quality in interracial relationships. When Lehmiller and Agnew (2006) explored the association between relationship marginalization and relationship investment and commitment, they found that being in a marginalized relationship (i.e., same-sex, interracial, or age-gap) was associated with lower levels of investments in relationships. Stigma as a result of relationship type has been related to lower levels of commitment, trust, love, and even relationship satisfaction (Baptist et al., 2019; Vazquez et al., 2019). There is evidence that not all individuals in interracial relationships are negatively impacted by stigma. The extent to which individuals hold egalitarian values and perceive the couple as having strong coping skills can dampen the negative impact of stigma on relationship quality and wellbeing (Rosenthal et al., 2019). Another important element to the existing conclusions of stigma and relationship functioning is participants' perception of their marginalized status and the attribution of experiences of discrimination to their relationship type. Implicit in these studies is the acknowledgment that individuals vary in the extent to which they perceive marginalization and experience discrimination as part of an interracial couple. Though this research establishes the connection between stigma and relationship quality in interracial relationships, there has not been a substantial investigation into what contributes to the individual differences in perceptions of relationship stigma; differences might be attributed to group memberships, both at the individual and dyadic levels.

People of color experience and are more aware of race-based discrimination than White individuals (Pinel, 1999; Tran et al., 2017). This difference has been demonstrated in academic (Guzman et al., 2016; Leath and Chavous, 2018) and health contexts and has been associated with negative mental and physical health outcomes (Ong et al., 2017; James, 2020). Furthermore, there is evidence that the racial composition of a relationship has an influence on the occurrence of interracial relationships (Livingston and Brown, 2017). The rate of occurrence may be related to the stigma that a couple experiences. Black interracial relationships are the least frequent of relationships between BIPOC and White partners (Livingston and Brown) and report the poorest relationship quality (Kroeger and Williams, 2011). Though informative, this research is limited in the ability to address the expanse of felt stigma in interracial relationships. Specifically, data that indicate differences in stigma between BIPOC and White people in interracial relationships may inform perceptions of the stigma of BIPOC-White relationships but are less informative in BIPOC interracial relationships. Additionally, conclusions regarding the racial composition of the couple are indirect and especially nuanced as other group membership differences (such as gender) also inform perceptions or prevalence (Livingston and Brown, 2017).

### Racial-Ethnic Worldview

Understanding heterogeneity in perceptions of stigma can be advanced with a novel understanding of the impact of race that is not predicated on ascribed group membership. Brooks et al. (2021b) introduced the concept of a racial worldview (here termed Racial-ethnic Worldview) as a way of assessing the impact of race on the perceptions and experiences of individuals. Racial-ethnic worldview (REW) suggests that race has an impact on the lived experiences of individuals across the (1) intrapersonal, (2) interpersonal, and (3) institutional domains of life. In its conceptualization, REW is understood as an individual difference variable that reflects the unique constellations of one's understanding of the importance of race and ethnicity. The three domains of REW are understood to be unique, though perhaps interdependent, such that each can be understood as having its own distribution. Rather than be understood as a composite and reduced to a single value or investigating the association between each domain separately, REW is assessed using a person-centered approach. Unlike variable-centered approaches that assume that a population is homogenous, person-centered approaches assume that a population is made of subpopulations [or REW types in the context of the current study; (Howard and Hoffman, 2018)]. As a consequence, REW offers a holistic perception of how the three domains interact within a population to form subpopulations of individuals with shared characteristics. An additional benefit of a typology in Racial-ethnic Worldview is that it allows for the classification of individuals in such a way as not to conclude that one type is “better” than the other, but rather allows for an exploration of the influence of Racial-ethnic Worldview on specific outcomes. Knowing how one group or Racial-ethnic Worldview differs from another or what differences matter has implications for relationship science in diverse contexts.

At the institutional level, REW assesses the extent to which a person understands race as part of a social power hierarchy which affects individuals' access to institutional resources (e.g., healthcare, housing, and financial systems). The system of racism is understood to have a more deleterious effect on the life experiences of BIPOC than on White people. The institutional understanding of race is not unlike critical consciousness scholarship such as critical race theories in that it directly assesses ones understanding of power and racism within the US (Delgado and Stefancic, 2017). In its current conceptualization, REW assesses the awareness of the institutional influence of race through the work of (Neville et al., 2000) on color-blind racial ideology (CBRI). CBRI upholds the existing race-based power structure and access to means that are embedded in the current social systems. The conceptualization of CBRI as a legitimizing ideology has received challenges from some (Locascio, 2014; Locke, 2014), but recent scholarship has demonstrated that the adoption of CBRI is an attempt to evade an acknowledgment of the systemic racism that BIPOC experience that is different than attempts at color-evasion (Mekawi et al., 2020).

At the interpersonal level, REW is understood as the extent to which an individual values ethnic or racial group differences and perceives those differences as beneficial to society. It is assessed through Berry and Kalin's (1995) operationalization of multiculturalism. Especially important to the conceptualization of the interpersonal domain is an absence of the evaluation of intergroup bias and explicit overtures to group-based hierarchies or power differentials. This is markedly different than social dominance orientation which favors a group-based hierarchy (Kteily et al., 2017). The interpersonal domain of REW mirrors aspects of egalitarianism or pluralism (Berbrier, 1998) because it is not-hierarchical and ascribes value to racial or ethnic group membership. It also extends on these frameworks in that it highlights the interpersonal and societal benefit of cross-group interactions and mutual influence among groups; a facet that is not explicitly captured in egalitarianism or pluralism.

Finally, at the intrapersonal level, REW is an assessment of the personal meaning and evaluation of group membership or identity. Similar to the interpersonal domain, the intrapersonal domain is not embedded with racial power dynamics such as privilege, oppression, internalized racism, or animus toward an outgroup. Additionally, it is conceptualized to be commensurate across group memberships. As outlined in existing models/theories of racial identity, healthy identity among people of color may involve deprogramming internalized notions of white supremacy (Helms, 1990). This process may involve adverse reactions to White people, include moments of self-disapproval, and may result in an experience of strong racial pride (sometimes accompanied by an oppositional stance to whiteness; Cross, 1995). Conversely, White racial identity models suggest feelings of guilt, shame, and anger about White racial group membership as one grasps notions of white privilege or being the beneficiaries of racist policies (Helms, 1995). These qualitatively different processes challenge the utility of framing the intrapersonal impact of race as one of racial identity. The use of racial identity is further complicated by the embedding of power in racial identity models (i.e., racial hierarchies regarding access). As a construct intended to be commensurate across group membership, Racial-ethnic Worldview operationalizes the intrapersonal domain as an ethnic identity.

Scholars have opined about the conflation of race and ethnicity and their measurement in scholarship. It is understood that whereas both are social constructs, the race is usually group membership-based, in large part, on physical characteristics such as skin color, phenotype, hair texture, etc. and ethnicity alludes to culture, values, language, customs, etc. Though outside of the scope of the current project, scholars have discussed the conflation in the measurement of racial and ethnic identity (Cokley, 2007; Phinney and Ong, 2007). The two constructs are treated as interrelated within broader society as well as within research (e.g., the proliferation of the use of terms such as racial-ethnic and ethno-racial in literature exploring identity and race within relationships). The current conceptualization of the Racial-ethnic Worldview utilizes the Multigroup Ethnic Identity Measure (Phinney, 1992). It has been shown to be unrelated to reports of race-based power structures (experiences of institutional and individual racism; Helms, 2007) and has shown strong psychometric properties across group membership. By conceptualizing the intrapersonal level as ethnic identity, the Racial-ethnic Worldview can account for how people make meaning of group membership without allusions to power and/or racial hierarchies.

By assessing individuals in each of the domains of Racial-ethnic Worldview, researchers are able to differentiate subgroups within a sample of individuals using scores on the identified measures. Though theoretically, each person has a unique combination of values, grouping similar response patterns can allow for an examination of subgroup differences. For example, researchers have differentiated those who deny institutionalized racism from those with favorable intergroup attitudes (e.g., color-blind types from multiculturalist types) and used scores on the intrapersonal domain to identify three types of color-blind REW. Specifically, those who felt positively about their ethnic identity and have engaged in behaviors to learn more about their ethnicity were classified as Color-blind Achieved and those who did not engage in high levels of exploration but still had favorable feelings were classified as Color-blind Affirmed. Finally, a third group, who tended to deny institutionalized racism and had negative feelings about their ethnic identity, were named Color-blind Unaffirmed (Brooks et al., 2021b). This classification system has been used to explore differences in the reported experiences of individuals in interracial relationships.

#### Racial-Ethnic Worldview and Interracial Relationships

A developing body of research has shown that Racial-ethnic Worldview impacts whether partners in interracial relationships initiate discussions about race in their relationship (Brooks et al., 2021a). Brooks et al. (2021b) classified individuals in interracial relationships with those who had a strong understanding of institutional, interpersonal, and intrapersonal aspects of race as Multiculturalists, and subdivided those with a relatively lesser understanding of institutionalized racism and poorer intergroup attitudes as Color-blind types. They concluded that those who (a) deny institutional forms of racism (b) are less certain of the intrapersonal meaning of race or ethnicity, and (c) have poorer interpersonal attitudes toward other groups (Color-blind types), were more likely to report not discussing race. On the other hand, issues of discrimination and systemic racism were more likely to be discussed by interracial partners with (a) a strong intrapersonal sense of ethnicity, (b) positive intergroup attitudes, and (c) who were less likely to deny institutional forms of racism and racial privilege (Brooks et al., 2021a). Pertinent to the current study, Racial-ethnic Worldview has implications for the perception and awareness of stigma as well. A recent study has connected a particular type of Racial-ethnic Worldview with greater perception and attribution of racism based on relationship type. Specifically, those that both minimize or dismiss the occurrence of racism and also hold below-average levels of positive affect for their identity experienced the greatest amount of discrimination and marginalization (Brooks, 2022b). Collectively, the existing scholarship on REW supports the use of a person-centered approach as the conclusions drawn was only reached when the interaction among domains was assessed.

The current research offers an explicit exploration of the associations between Racial-ethnic Worldview, stigma, and relationship quality. It expands on prior research not only through the direct investigation into relationship quality but also uses more sophisticated analyses to support the utility of the use of a classification system using a Racial-ethnic Worldview.

Prior research has connected felt stigma and experiences of marginalization with poorer relationship outcomes (Lehmiller and Agnew, 2006; Rosenthal and Starks, 2015). Brooks (2022b) found that those who denied institutionalized racism and had poor intrapersonal feelings regarding their ethnicity reported greater stigma. As such, it is plausible that REW may be directly associated with reports of relationship quality. Because we use an alternative person-centered analysis to classify participants (latent profile analysis) than has been used in prior research (*k-*means cluster), it is possible that the REW types that are identified within the current data do not replicate from prior studies. Nonetheless, we explore differences in relationship quality based on REW but are not able to offer a priori distinctions regarding which REW types may differ in relationship quality.

We explore the following question regarding the association between REW and relationship quality:

*Research Question 1:* Are there group differences in reports of relationship quality based on the Racial-ethnic Worldview?

As an expansion of previous research, we explore the association between relationship stigma, Racial-ethnic Worldview, and relationship quality. We hypothesize that the impact of stigma on relationship quality is moderated by a Racial-ethnic Worldview. Similar to RQ1, because of the novelty of this person-centered approach, we are not able to offer specifically a priori distinctions. However, it is plausible that acknowledging issues of institutional racism may benefit individuals in interracial relationships as they are more likely to discuss interpersonal forms of racism and individual discrimination with their partners (Brooks et al., 2021a). This may lead to better perceptions of quality as partners may be able to support each other.

*Hypothesis 1a:* The association between relationship stigma received from family and reports of relationship quality will be moderated by the Racial-ethnic Worldview.
*Hypothesis 1b:* The association between relationship stigma received from the public and reports of relationship quality will be moderated by the Racial-ethnic Worldview.
*Hypothesis 1c*: The association between relationship stigma received from the friends and reports of relationship quality will be moderated by the Racial-ethnic Worldview.

## Methods

### Participants

Participants were recruited from Amazon Mechanical Turk (MTurk) as well as from a large midwestern university. Across recruitment efforts, there were 856 respondents. Of initial respondents, 520 did not qualify for the study based on their responses to screening questions (e.g., they were not currently in a romantic relationship, they were not currently in a relationship with someone of a different race, or they were not based in the United States). Potential participants were informed of eligibility requirements prior to completing the survey and if excluded because of inclusion criteria were prevented from attempting the study again. An additional 23 were removed for incomplete responses, of which 7 stopped the survey prematurely and 16 were prevented from completing the study because of missed attention checks. The final sample of participants was 313 individuals who indicated that their race was different than the race of their partner of at least 6 months, was at least 18 years old at the time of completion of the study, and resided in the United States. Supplementary Table 1 shows a comparison of the sample demographics and key variables. Across both samples, participants' average age was 28.69 years (*SD* = 9.29). Most participants, 55.6%, identified as female (*n* = 174); 43.1% (*n* = 135) identified as male, and 1.3% (*n* = 4) identified as non-binary. Roughly 8% of the sample (*n* = 25) indicated that they were in a same-gender relationship and the remaining participants (*n* = 278) indicated that they were in a different-gender relationship. The sample was racially diverse with 55.6% (*n* = 174) of participants identifying as White, 14.7% (*n* = 46) identifying as multiracial, 11.8% (*n* = 37) identified as Black and 9.6 and 7.3% identifying as Latinx (*n* = 30) or Asian (*n* = 23), respectively. Three participants identified as Native American. Participants included 55.6% (*n* = 174). White participants reporting their relationship with a BIPOC partner, 28.4% (*n* = 89). BIPOC participants reporting their relationship with a White partner, and 16.0% (*n* = 50). BIPOC participants reporting their relationship with a BIPOC person of a different racial background than themselves.

### Procedure

A Freedom of Information Act (FOIA) request was completed to obtain the email addresses of students, faculty, and staff at a Midwestern university. An email invitation to the survey was sent to the distribution list indicating the eligibility criteria (18 years or older, been in an interracial relationship for the past 6 months or more). An incentive of being entered into a raffle for one of four $50 gift cards was offered. For MTurk, the link to the survey was posted in a Human Intelligence Task (HIT) for eligible workers to complete. The brief description of the HIT was listed as a study of relationship quality. The extended description and an informed consent document outlined the study including the nature of responses and inclusion criteria. For both the MTurk and university sample, participants completed a brief screener for age and interracial relationship status to ensure eligibility for the study. Participants from both samples had the same inclusion criteria and were at least 18 years old, currently in a relationship with someone of a different race for 6 months, and living in the United States. Participants reported their race and the race of their partner separately and were asked if they identified the relationship as an interracial relationship. Those who did not identify as interracial or reported that they and their partner were of the same racial group were removed from the study. The study measures were presented in a random order to study participants. For the university sample, at the end of the survey, to keep participants' survey responses separate from the information needed for the raffle, participants were provided a link to a new survey to enter their email addresses. MTurk workers were paid $1.06 after successfully completing the survey and passing the required attention and manipulation checks.

### Measures

#### Institutional Domain: Color-Blind Racial Ideology

Participants' perceptions of the institutional impact of the race were assessed using the Color-blind Racial Attitudes Scale (CoBRAS; Neville et al., 2000). The 20-item measure contains 3 subscales: Racial Privilege—unawareness of White Privilege, Institutional Discrimination—an unawareness of discrimination at the institutional level, and Blatant Racial issues—an unawareness of racial discrimination. Items on the CoBRAS are rated on a 6-point Likert-type scale ranging from *strongly agree* (1) to *strongly disagree* (6), with items summed to create a total score where higher scores indicate a greater level of colorblindness. The initial construction and validation of the CoBRAS demonstrated good psychometric properties with reliability estimates ranging from 0.84 to 0.91(Neville et al., 2000). In the current study, reliability estimates for the CoBRAS total score were α = 0.94. Example items include statements such as “Racism may have been a problem in the past, but it is not an important problem today” and “Racial problems in the US are rare, isolated situations.”

#### Interpersonal Domain: Multicultural Ideology

Participants' perceptions of the interpersonal impact of the race were assessed using their scores on the Multicultural Ideology (MCI) scale (Berry and Kalin, 1995). The ten items of the MCI are measured on a 5-point Likert-type scale ranging from *strongly disagree* (1) to *strongly agree* (5) designed to assess participants' beliefs about cross-cultural interactions. Items averaged with higher scores indicate a greater valuing of a diverse society with multiple unique cultures. The MCI has been shown to have good reliability in international populations with Cronbach's α estimates ranging from 0.82 to 0.88 (Verkuyten and Brug, 2004) as well as with samples in the United States, 0.82–0.85 (Brooks and Neville, 2017). The reliability estimate for the current sample was α = 0.9. An example item is “We should recognize that cultural and racial diversity is a fundamental characteristic of United States society.”

#### Intrapersonal Domain: Ethnic Identity

The Ethnic Identity Scale (EIS; (Umaña-Taylor et al., 2004)) was used as a way of measuring the influence of race at the intrapersonal level. The EIS is widely used as an assessment of three domains of ethnic identity and is informed by the work of Marcia (1980) as cited by Umaña-Taylor et al. (2004) and Phinney (1989). The three independent dimensions of the EIS are exploration, “I have read books/magazines/newspapers or other materials that have taught me about my ethnicity,” resolution, “I am clear about what my ethnicity means to me,” and affirmation “I dislike my ethnicity,” reverse scored. The 17 items of the EIS are measured on a 4-point Likert scale from *Does not describe me at all* (1) to *Describes me very well* (4) with items summed to greater a total score on each subscale, where higher values indicate more exploration, resolution, and affirmation. Initial construction of the EIS showed good reliability estimates for each dimension (exploration α = 0.89–0.91, resolution α = 0.89–0.92, and affirmation α =0.84–0.86). In the current study, reliability estimates for each subscale were good with the following results: affirmation α = 0.91, exploration α = 0.89, and resolution α = 0.87.

#### Stigma

To assess participants' experiences of scrutiny because they are in an interracial relationship, the Relationship Stigma Scale (RSS; Rosenthal and Starks, 2015) was administered. The scale explores negative experiences generated from family (“family members do not acknowledge your relationship and refer to your partner as your ‘friend”'), friends (“friends make comments about your partner and relationship that offend you”), and the public (“People are rude to you/give you an attitude”) more generally. Respondents report on the frequency at which they have experienced each item ranging from 1 *never* to 4 *often*. Reliability estimates for each subscale were public α = 0.87, family α = 0.61, and friends α = 0.6. The RSS was presented using the original format which contained two different scales used across the 20 items. Following the authors' scoring procedures, we used standardized scores to report experiences of stigma across each source of scrutiny as *Z*-scores, independently, where higher scores indicate more than average experiences of stigma for the sample. As in the original study, we used the unaltered form of the RSS which was administered to both interracial and same-sex partners.

#### Relationship Quality

Participants also completed the Perceived Relationship Quality Components Inventory (Fletcher et al., 2000). Designed to assess participants' feelings of love, passion, trust, commitment, intimacy, and satisfaction, the six subscales of the PRQCI each contain three items on a *not at all* (1) to *extremely* (7) scale. Sample items include “How committed are you to your relationship” or “How satisfied are you in your relationship.” Higher scores indicate greater relationship quality. In the current study, we were interested in the global relationship quality of participants and followed the suggestion of Fletcher et al. (2000) to retain the first item of each subscale as an overall assessment of relationship quality. Using the six items resulted in good reliability, α = 0.87, a score that we refer to as relationship quality.

This study is part of a larger data collection effort and was not pre-registered. The data on which the research is based is available from the first author. The execution of this research received human subjects approval from the local Institutional Review Board.

## Results

Table 1 contains the zero-order correlations among the study variables. There were small to moderate correlations among the key variables of the study, with a strong inverse association identified between the institutional domain as measured by the CoBRAS and the interpersonal domain measured by the MCI.

**Table 1:** Zero-order correlations.

	**1**.	**2**.	**3**.	**4**.	**5**.	**6**	**7**.	**8**.	**9**.
1. Relationship quality	–								
2. Stigma from public	−0.11*	–							
3. Stigma from family	−0.17**	0.53**	–						
4. Stigma from friends	−0.27**	0.56**	0.69**	–					
5. Color-blind racial ideology	−0.22**	−0.10	−0.07	0.16**	–				
6. Ethnic identity: affirmation	0.13*	−0.29**	−0.28**	−0.44**	−0.01	–			
7. Ethnic identity: exploration	0.12*	0.17**	−0.07	0.07	−0.10	0.09	–		
8. Ethnic identity: resolution	0.11	−0.01	−0.06	−0.09	−0.07	0.22**	0.55**	–	
9. Multicultural ideology	0.27**	0.03	0.04	−0.18**	−0.74**	0.07	0.20**	0.15**	–

*p < 0.05; **p < 0.01.

### Racial-Ethnic Worldview

**Figure 1:**
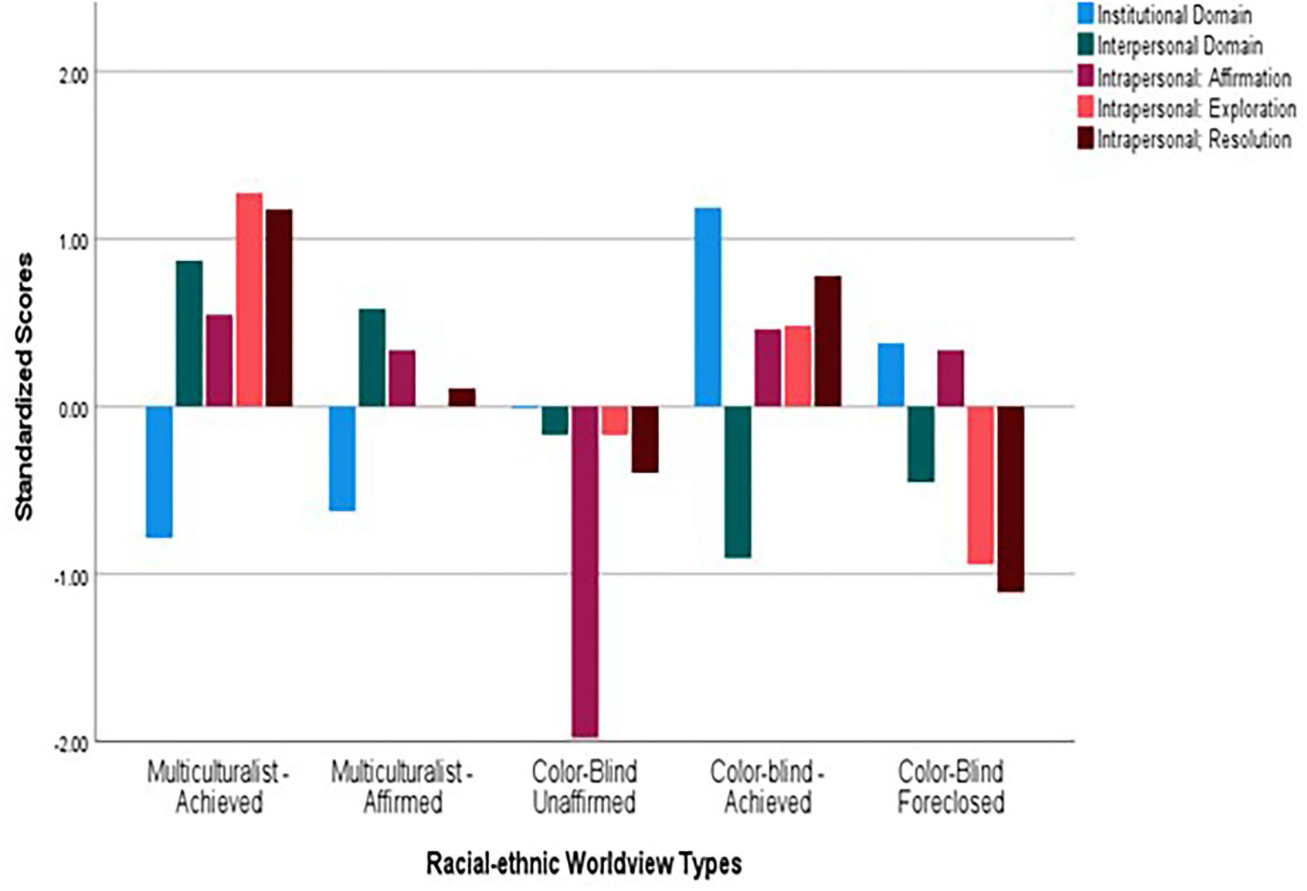
Racial-ethnic Worldview domains by type. Institutional Domain is assessed by scores on the Color-blind Racial Attitudes Scale (CoBRAS; Neville et al., 2000) higher scores indicate a greater denial of institutionalized racism. Interpersonal Domain is assessed by scores on the Multicultural Ideology Scale (Berry and Kalin, 1995) higher scores indicate a more positive intergroup attitude/ The Intrapersonal Domain is assessed using the Ethnic Identity Scale (Umaña-Taylor et al., 2004). Higher scores indicate more positive affect (affirmation) greater exploration of ethnic identity (exploration) and greater sense of commitment to ethnic identity (Resolution).

To classify participants based on the Racial-ethnic Worldview, mixture modeling techniques were employed using the MCLUST package of R (latent profile analysis; Scrucca et al., 2016). Intrapersonal (ethnic identity exploration, resolution, and affirmation), interpersonal (multicultural ideology), and institutional (CBRI) understandings of the race were used as indicators. Using a proportional diagonal covariance matrix structure, a series of models with varying numbers of profiles were identified. Though results indicated that minimization for all indices was reached at the enumeration of six profiles (see Supplementary Table 2), further examination suggested model overfit as one profile contained only *n* = 10 participants. By all indicators, the five-profile solution provided a better fit to the data than the 4-profile solution (which was explored based on the four-cluster solution reported in Brooks et al., 2021b). Given the robust indication of better fit and that the 5-profile solution provided comparable distinction of clusters as the 4-profile solution, entropy = 0.87 and 0.85, respectively, the 5-profile solution was interpreted. Figure 1 depicts the standardized means of the indicators for each profile in the 5-profile solution.

There were four of the five identified profiles which reproduced the four clusters of the *k-*means solution reported in Brooks et al. (2021b). A total of three groups (the color-blind types) were characterized by a relatively high denial of the institutionalized impact of the race compared to less positive scores on the interpersonal domain and various levels of intrapersonal understanding of race. A Color-blind Achieved profile represents participants who more readily deny the institutional impact of race and who hold a positive intrapersonal understanding of race (*n* = 55). Though the Color-blind Foreclosed profile reported positive affiliation with their ethnic group, they exhibited little exploration of this identity (*n* =70). The third Color-blind profile, Colorblind-Unaffirmed is most distinctively characterized by its low level of affirmation which indicates relatively low levels of positive feelings in the intrapersonal domain (*n* = 52). In addition to the color-blind types that were reported in Brooks et al. (2021b), the Multiculturalist type is characterized by relatively high scores in the interpersonal and intrapersonal domains, domain, and low denial of the institutional impact on race was also reproduced (*n* = 38). This group was classified as Multiculturalist Achieved in the current study. The final profile, identified as Multiculturalist Affirmed, is different than prior results and exhibits the same interpersonal and institutional understanding of race as the reproduced Multiculturalist type, but has engaged in relatively less exploration of their group membership and commitment to understanding the group (*n* = 98).

Table 2 displays the frequency of REW assignments based on racial group membership and gender. For the gender analysis, only those who identified as either a man or woman were included as too few identified as others to warrant meaningful comparisons. A chi-square test indicated a difference in prevalence of REW by race, $\chi_{\left(4\right)}^{2}$ = 25.04, *p* < 0.001. Using a Bonferroni correction to adjust for Type 1 error, follow-up analyses indicated that BIPOC individuals were more likely to be identified as Multiculturalist Achieved, χ^2^ (1) = 17.64, *p* < 0.01, while White participants were less likely to be classified as such. Furthermore, White participants were significantly more likely to be classified as Color-blind Foreclosed, $\chi_{\left(1\right)}^{2}$ = 9.86, *p* < 0.005, whereas BIPOC individuals were less likely to be classified as Color-blind Foreclosed. A parallel set of analyses were conducted to test for gender differences in REW classification. After a significant overall test, $\chi_{\left(4\right)}^{2}$ = 14.40, *p* < 0.01, follow-up analyses indicated that women were less likely to be classified as Color-blind Achieved, $\chi_{\left(1\right)}^{2}$ = 9.86, *p* < 0.005, whereas men were more likely to be classified as such. There were no differences in age in REW types, *F*_(4, 308)_ = 0.6, *p* > 0.05.

**Table 2:** Racial-ethnic Worldview by race and gender.

			**Participant race**			**Participant gender**		
			**White**	**BIPOC**	**Total**	**Man**	**Woman**	**Total**
Racial-ethnic Worldview	Multiculturalist—achieved	*N*	9	29	38	14	24	38
		Percentage of group	5.2%_a_	20.9%_a_	12.1%	10.4%	13.8%	12.3%
	Multiculturalist—affirmed	*N*	55	43	98	32	65	97
		Percentage of group	31.6%	30.9%	31.3%	23.7%	37.4%	31.4%
	Color-blind unaffirmed	*N*	33	19	52	21	30	51
		Percentage of group	19.0%	13.7%	16.6%	15.6%	17.2%	16.5%
	Color-blind—achieved	*N*	27	28	55	34	20	54
		Percentage of group	15.5%	20.1%	17.6%	25.2%_c_	11.5%_c_	17.5%
	Color-blind foreclosed	*N*	50	20	70	34	35	69
		Percentage of group	28.7%_b_	14.4%_b_	22.4%	25.2%	20.1%	22.3%
Total	*N*	174	139	313	135	174	309	
	Percentage of group	100.0%	100.0%	100.0%	100.0%	100.0%	100.0%	

*Percentages with a shared subscript differed significantly across columns. For example, there were more BIPOC individuals classified as Multiculturalist Achieved than expected based on sample frequencies. Conversely, fewer White participants were identified as Multiculturalist Achieved than expected*.

### Racial-Ethnic Worldview and Relationship Quality

To examine Research Question 1, whether there is a difference in reports of relationship quality by Racial-ethnic Worldview, a single ANOVA was conducted (see Table 3 for descriptive statistics). Homogeneity of variance was violated based on Levene's test (*p* = 0.006), therefore Welch's ANOVA and equal variances not assumed contrasts were used, with the effect size reported based on the unadjusted analyses. The results of the analysis indicated that there was a statistically significant difference in the reports of relationship quality based on Racial-ethnic Worldview, *F*_(4, 126.63)_ = 3.25, *p* = 0.01, unadjusted $\eta_{p}^{2}\;=$ 0.04. Games-Howell *post-hoc* analyses indicated statistically significant differences in relationship quality for Multiculturalist Achieved compared to Colorblind Unaffirmed with the Multiculturalist Achieved type reporting greater relationship quality than the Colorblind Unaffirmed type. While Multiculturalist Affirmed vs. Colorblind Unaffirmed demonstrated a similar trend (i.e., the Multiculturalist subtype reported greater relationship quality than the Colorblind Unaffirmed) it was not statistically significant, *p* = 0.06. No other pairwise comparison reached statistical significance.

**Table 3:** Descriptive statistics for relationship quality based on racial-ethnic worldview groups.

**Racial-ethnic Worldview group**	***n***	***M***	***SD***	**Min**	**Max**
Multiculturalist achieved	38	6.35^a^	0.77	4.33	7.00
Multiculturalist affirmed	98	6.25^ab^	0.68	3.67	7.00
Colorblind unaffirmed	52	5.80^b^	1.07	2.50	7.00
Colorblind achieved	55	6.02^ab^	1.04	2.67	7.00
Colorblind foreclosed	70	5.99^ab^	0.89	2.50	7.00

*Means that have no superscript in common are significantly different at the p = 0.05 level based on Games-Howell*.

#### *Post-Hoc* Analysis

In viewing the results of the REW classification, we explored two additional analyses. These *post-hoc* analyses were conducted using planned contrasts and collapsed across REW types. The first was among those who were classified as Multiculturalist type (Achieved and Affirmed) and those classified as Color-blind types (Achieved, Foreclosed, and Unaffirmed). The second was between those with achieved distinction (Multiculturalist and Color-Blind types) and the other three REW types. These contrasts indicated that there was a statistically significant difference in relationship quality when comparing the Multiculturalist REW types vs. Color-blind REW types, *t*_(183.91)_ = 3.44, *p* = 0.001, unadjusted *d* = 0.38; but not when comparing Achieved groups (Multiculturalist Achieved, Color-blind Achieved) vs. other groups (Multiculturalist Affirmed, Color-blind Unaffirmed, Color-blind Foreclosed), *t*_(169.59)_ = 1.47, *p* = 0.14, unadjusted *d* = 0.17. Taken together, these results suggest that there were significant differences in relationship quality across Racial-ethnic Worldviews.

### Racial-Ethnic Worldview Moderates the Association Between Stigma and Relationship Quality

Hypotheses 1a−1c held that a Racial-ethnic Worldview would moderate the relationship between relationship stigma and relationship quality. Three separate hierarchical models were conducted with relationship quality as the criterion; the main effects of Racial-ethnic Worldview and one of the three types of relationships stigma and their interaction term were used as predictors. To reduce the risk of type I error, a Bonferroni correction was made such that each overall regression model needed to have a probability of *p* < 0.017, to be considered statistically significant. Davidson-Mackinnon adjustments were made to correct for violation of homogeneity among Racial-ethnic Worldview types. Results indicated that the model predicting relationship quality from family stigma and Racial-ethnic Worldview was statistically significant, *F*_(9, 303)_ = 3.29, *p* < 0.001, Adj *R*^2^ = 0.14. Because Racial-ethnic Worldview differences in relationship quality were reported in RQ1, we report here on the interactions. The interaction term was significant, *F*_(4, 303)_ = 3.92, *p* < 0.01, *Adj R*^2^ = 0.07. With the exception of the Color-blind Achieved profile, *b* = −0.86 (0.39), *t* = –2.22, *p* < 0.05, the connection between family stigma and relationship quality did not differ from zero. The results of Hypothesis 1a suggest that the Color-blind Achieved type is uniquely impacted by family stigma for their relationship, such that the more stigma this type experiences from family members, the poorer their reports of relationship quality. The test of Hypothesis 1b concerning the interaction of Racial-ethnic Worldview and public stigma offered different conclusions. The overall model was significant, *F*_(9, 303)_ = 2.16, *p* < 0.001, Adj *R*^2^ = 0.08. The Color-blind Achieved interaction approached the threshold, *b* = −0.61 (0.33), *t* = −1.84, *p* = 0.06, suggesting that the Color-blind Achieved greater perception of stigma from the public was associated with poorer reports of relationship quality. For all other REW types, the relationship between public stigma and relationship quality was not significant. By contrast, the test of Hypothesis 1c offers different conclusions. Though the overall model was statistically significant, *F*_(9, 303)_ = 3.97, *p* < 0.001, Adj *R*^2^ = 0.11, the only unique REW contributor to the model was the contrast of Multiculturalist Achieved compared to Color-blind Foreclosed *b* = −0.37 (0.18), *t* = −1.99, *p* < 0.05. The comparison between Multiculturalist Achieved and Color-blind Unaffirmed was close to the threshold but not statistically significant, *b* = −0.39 (0.18), *t* = −1.93*, p* = 0.06. The conclusions from Hypothesis 3 suggest that, regardless of REW type, greater stigma experienced by friends was associated with poorer reports of relationship quality. The strength of this association was not different from any other REW compared to others. These results offer some support for the moderating effect of the Racial-ethnic Worldview on relationship quality. Specifically, those with a Color-Blind Achieved Racial-ethnic Worldview were especially impacted by stigma from family. However, for all Racial-ethnic Worldview groups, greater stigma from friends was associated with a decline in relationship quality.

## Discussion

The purpose of the current study was to offer a robust investigation into the associations between Racial-ethnic Worldview, relationship stigma, and relationship quality. Broadly, this investigation provides evidence that how individuals in interracial relationships consider the impact of race and ethnicity in the institutional, interpersonal, and intrapersonal domains has significant implications for their evaluation of the relationship and their susceptibility to the negative impacts of discrimination. The results of this study support emerging research on the utility of REW to differentiate among interracial relationship partners. Not only were previous REW types reproduced and refined, but further support was offered for unique vulnerabilities to stigma among some individuals, but not others. The results also expand the literature by demonstrating that there are consequences in relationship quality based on REW.

The results from the study add to the literature concerning Racial-ethnic Worldview. Using the same indicator variables as within the current study, Brooks et al. (2021b) introduced the concept of Racial-ethnic Worldview using k-means clusters and reported a four-cluster solution. Here using a model-based classification system, we found the same four classes of responses plus a fifth that further differentiated among individuals with a multiculturalist perspective. The replication of those original four-classes and the use of the more statistically sound mixture analyses, which are less prone to spurious results and model overfitting, suggests that there is great utility in the person-centered approach. Our analyses produced an empirically identified fifth Racial-ethnic Worldview not originally identified in Brooks et al. (2021b). The Multiculturalist Affirmed class suggests that there is a subset of multiculturalist views that have a relatively less critical or extensive exploration of their identity though they still have positive feelings toward it. Though not distinct from each other, the Multiculturalist Affirmed and Achieved groups reported better outcomes than the Color-blind types.

The investigation of Research Question 1 offered a direct test of an association between REW and relationship quality, whereas prior studies have theorized about possible connections only indirectly (Brooks, 2022b). The results concluded that participants' REW is associated with relationship quality. The pairwise analyses indicated that those who were more conscious of institutional racism had positive intergroup attitudes, and held a positive intrapersonal understanding of race (Multiculturalist Achieved) reported greater relationship quality than those who more readily denied the institutional influence of race, had a less positive interpersonal score and a poorer intrapersonal understanding (Color-blind Unaffirmed). The mechanisms by which these conclusions are reached may be 2-fold. First, prior research has concluded that the Color-blind Unaffirmed profile (one of the original clusters reproduced in the current study) reported the greatest amount of stigma from all three domains of public, family, and friends of all clusters (Brooks, 2022b), combined with established research that demonstrates the impact of marginalization and stigma on relationship outcomes (Lehmiller and Agnew, 2006), it can be concluded that felt or experienced stigma may depress reports of relationship quality within this profile. Secondly, the implied positive impact of a more favorable intrapersonal understanding of race (as captured in the Multiculturalist Achieved profile) mirrors prior research that strong racial identity is associated with greater satisfaction in interracial relationships (Leslie and Letiecq, 2004). In fact, the Multiculturalist Achieved profile had the highest reports of identity exploration and resolution, distinguishing it from most other profiles. In this way, a stronger and more positive identity was again shown as beneficial for relationship outcomes.

The *post-hoc* comparisons of Research Question 1 suggest that participants' understanding of interpersonal and institutional impacts of race also inform relationship quality. The two Multiculturalist types reported greater relationship quality than the three Color-blind types, suggesting that the extent to which participants reflected a critical understanding of power by race within a US context, and still valued group differences as beneficial for society, positively impacted their experiences in their relationship. This conclusion is especially illuminating considering prior research on REW. Mixed-method analyses have indicated that some color-blind types are less likely to discuss race openly with their partner (Brooks et al., 2021a) and that Multiculturalist types are more likely to discuss issues of systemic racism and efforts to mitigate its impact (i.e., social protest movements; Brooks et al., 2021a). This is especially encouraging as prior research has found that these conversations can be tense or dismissive of one person's experience (Killian, 2002), or even limited to understanding the difference in the race as inherently problematic (Brummett, 2017).

Regarding Hypotheses 1a−1c, using separate hierarchical analysis, we found that REW moderated the association between family stigma and relationship quality. Specifically, for one profile, the Color-blind Achieved, greater stigma from family was associated with poorer relationship quality, and a similar pattern was emerging for perceptions of public stigma. Among the five profiles, Color-blind Achieved reported the strongest denial of the institutional impact of race and the least positive response in the interpersonal domain. Though they demonstrate a great aversion to the discussion of race in interpersonal and institutional domains, intrapersonal meaning and connection are exceeded only by the Multiculturalist Achieved profile. Though the current study cannot conclude definitively why Color-blind Achieved profiles are especially sensitive to stigma, there are several possibilities. Perhaps those with this REW struggle with a way of framing or understanding the opposition to their relationship and perceive the stigma as a personal attack. Or it may be that the frustration of dealing with stigma may have deleterious effects on functioning. Stated differently, perhaps there is a benefit to relationship quality of acknowledging racism or valuing group differences that the Color-Blind Achieved group is not able to draw upon. For example, experiences of discrimination can be externalized if one's REW acknowledges racism. Furthermore, perceiving that all groups are of value to society also offers a counter-narrative to the racial animus that is expressed toward the self or their partner. Alternatively, there is the concept of stigma consciousness, as this particular REW type becomes more aware of and experiences discrimination based on relationship type, they lack a structure to understand the racism that they experience. The Color-blind Achieved group may especially be susceptible to this dynamic as they have the highest denial of institutionalized racism. In not acknowledging racism the Color-blind Achieved group may not be able to have productive conversations with their partner that may serve as buffers against negative experiences. Qualitative investigations into exploring how interracial couples discuss race suggest that shared meaning or understanding is important. Among same-gender interracial couples, race and sexuality-related stress can lead to shared meaning-making whereby partners reframe negative experiences (such as stigma) as learning opportunities that enriched the partners. There is also evidence that couples engaged in direct problem-solving activities such as confronting racist or discriminatory treatment (Rostosky et al., 2008).

In any case, the influence of the public and family are the only domains in which the dynamic presents. There were no differences in the effect of stigma from friends on relationship quality, friend stigma had a consistent and comparable negative effect on relationship quality. Unlike family and the public more broadly, our friend groups are self-selected and thus objections from this group may be especially difficult to reconcile regardless of one's understanding of race and ethnicity.

### Implications

The conclusions of this study have implications for partners in interracial relationships as well as counselors. It appears that a critical and multidimensional understanding of race and racism informs subjective experiences in relationships. Prior research has shown that REW affects what impact individuals believe race has on their interracial relationships as well as what specific topics are discussed (Brooks et al., 2021a,b). The existing research could not directly assess whether there were impacts of these discussions on relationship functioning, the current study takes a step closer to understanding the implications of these conversations. In previous studies, the Multiculturalist clusters were more likely than others to discuss race and the current study finds that they have greater relationship quality. It may be that not just what is being discussed in relationships, but how those discussions are happening is important for relationship quality. Regarding the practice of couples counseling, it may prove beneficial to foster a critical consciousness regarding race and racism within interracial relationships that acknowledges and values the unique contributions of groups, the structural impacts of the race on lived experiences, and at least a positive effect on group membership. These are the qualities shared by those of the multiculturalist profiles. We suggest that the fostering of positive identification with group membership also coincides with recognition of racism and valuing of groups because holding a positive group membership feeling but also denying racism and devaluing other groups as beneficial may leave partners vulnerable to experiences of stigma (Brooks, 2022b).

We know that there are higher rates of dissolution for interracial couples (Bratter and King, 2008), and the results from this study suggest that there are classes of interracial relationship partners that are more susceptible to poorer relationship outcomes. Collectively, multiculturalist types may be especially resilient to stigma. Conversely, those who deny institutional racism and have poor intergroup attitudes are especially susceptible to opposition from the public and family. Of this latter group, either they perceive greater opposition than others (i.e., Color-blind Unaffirmed; Brooks, 2022b) or are more strongly negatively impacted by it than any other class of REW (i.e., Color-blind Achieved). This suggests, within the realm of therapy, that promoting multiculturalist worldviews among partners in an interracial relationship will be especially useful for the subjective experiences of partners. This would also require counselor awareness in regard to structural issues of racism and the benefit of a positive identity.

Ultimately, we find that REW is a particularly useful construct for understanding relationship quality for individuals in interracial relationships. Specifically, the person-centered approach is valuable for the study of interracial relationships. Approaches such as *k* means and latent profile analyses capture the nuance of the interactions among indicator variables that may be complicated by or go unseen within variable-centered approaches. For example, in the current study, the benefits to relationship quality of a strong and positive intrapersonal domain were only evidenced for those who also acknowledged the institutional impact of race and who had positive intergroup attitudes. The Color-blind Achieved, who showcased a robust intrapersonal identity, did not evidence the same boost in relationship quality as the Multiculturalist Achieved group. In fact, results from Hypothesis 1 suggest a comprehensive intrapersonal meaning was a liability for those who denied institutional racism. To reach similar conclusions using a variable-centered approach might include a multiple regression with five independent predictors and a five-way interaction term. The classification process of latent profile analysis is more parsimonious as the indicators increase and are not subject to arbitrary cutoffs that may lead to researcher defined groups that demonstrate no differences in relationship quality or do not reflect more organically occurring subpopulations; our results found no Multiculturalist Unaffirmed group, but a researcher defined grouping scheme may have created such a group.

### Limitations

The study is not without its limits, most explicitly is that the sample is not representative of the racial make-up of the United States. Though the conceptualization of REW allows for particular types to be espoused across groups, more representative samples are needed to ensure that the typology is indeed commensurate across racial group membership. However, the use of the person-centered Racial-ethnic Worldview provides a more meaningful examination of the impact of race than racial group membership because of the retention of ideological diversity. There is evidence that racial group membership and REW are not independent or inseparable. A higher percentage of BIPOC individuals were identified as Multiculturalist Achieved than White individuals and a higher percentage of White participants were identified as Color-blind Foreclosed than BIPOC participants. These results are to be expected. It is likely that racial group membership is a crude way of capturing racial socialization experiences. BIPOC individuals are more likely to experience explicit racial socialization messages concerning group membership and issues of racism than White individuals (Umaña-Taylor and Hill, 2020). As such the likelihood that they would have a stronger intrapersonal domain and are less likely to deny institutionalized racism is to be expected. However, given that classification in the Multiculturalist Affirmed group was not impacted by racial group membership, and that two Multiculturalist types were indistinguishable cv regarding the impact of stigma and reports of relationship quality, the implications of racial differences in the rate of Multiculturalist Achieved may be largely inconsequential in the current study. Similarly, the higher classification rate of White participants into the Color-blind Foreclosed type likely has little influence on the conclusions of the current study and is consistent with the above-cited trends of racial socialization.

Additionally, though a substantial amount of variance in relationship quality was accounted for by REW, there are some limits in the study design. Data were collected from only one partner in the relationship and the study was cross-sectional. Prior research has shown that there are partner effects on relationship quality in interracial relationships (Brooks et al., 2018), and having information from all partners in the relationship could allow for a greater understanding of the importance of Racial-ethnic Worldview at a dyadic level. Similarly, a longitudinal investigation would allow for temporal presence to establish causal connections between Racial-ethnic Worldview and relationship quality. Finally, the reliability statistics for the stigma from family and the public were below preferred standards which detracts the power of the analyses.

### Future Research

Of particular interest for future research is exploring dyadic and longitudinal variations. Such inquiries can address the Racial-ethnic Worldview as a dynamic process and explore potential changes in the Racial-ethnic Worldview and subsequent implications for relationship quality. Indeed, because the components of the Racial-ethnic Worldview are ideological, they may be subject to change over time and under various social conditions. For example, greater exploration of identity may increase affirmation among some interracial relationship partners. There may also be the possibility for manipulations that allow for increased awareness of the institutional effects of racism. Having data from all partners in the relationship can allow for the exploration of the impact of lack of agreement in worldview on relationship functioning. Additionally, the use of mixed-method investigations that explore how interracial relationship partners address feelings of marginalization based on the Racial-ethnic Worldview can provide empirical support for some of the conclusions or predictions made here.

### Conclusion

Though conducted in a WEIRD society, the current study is able to expand the understanding of interracial relationships by situating the inquiry with attention to the sociopolitical context of race and ethnicity within the United States. The use of the critical and comprehensive framework of REW offers insights into the diversity of thought and the implications therein for partners in interracial relationships.

## Data Availability Statement

The raw data supporting the conclusions of this article will be made available by the authors, without undue reservation.

## Ethics Statement

The studies involving human participants were reviewed and approved by Tennessee State University - Institutional Review Board. The patients/participants provided their written informed consent to participate in this study.

## Author Contributions

JB contributed to the conceptualization and design of the study, and wrote first drafts of the manuscript. JB and MM facilitated the collection of data and data management, and were each involved in data analyses and writing of results. MM read the manuscript for initial revisions. Both authors were involved in the revisions and editing of the subsequent manuscript and approved the final submission.

## Conflict of Interest

The authors declare that the research was conducted in the absence of any commercial or financial relationships that could be construed as a potential conflict of interest.

## Publisher's Note

All claims expressed in this article are solely those of the authors and do not necessarily represent those of their affiliated organizations, or those of the publisher, the editors and the reviewers. Any product that may be evaluated in this article, or claim that may be made by its manufacturer, is not guaranteed or endorsed by the publisher.
