# Supplementary material for: Stigma and Relationship Quality: The Relevance of Racial-Ethnic Worldview in Interracial Relationships in the United States
Source: Front Psychol. 2022 Jul 12;13:923019. doi: 10.3389/fpsyg.2022.923019 (PMC9315430; doi:10.3389/fpsyg.2022.923019)
Supplement: Supplementary file 1 [file Data_Sheet_1.docx]

**Table 1**

*Demographic and Variable Statistics by Sample*

|  |  | Sample | |
| --- | --- | --- | --- |
|  |  | University (*n* = 213) | MTurk (*n* = 100) |
| Demographics |  |  |  |
|  | Men | 35.7% | 59% |
|  | Women | 62.4% | 41% |
|  | Other | 1.9% |  |
|  | Age *M(SD)* | 28.24 (10.33) | 29.66(6.49) |
|  |  |  |  |
|  | Asian | 5.6% | 11% |
|  | Black | 8.9% | 18% |
|  | Latinx | 11.3% | 6% |
|  | Native American | .5% | 2% |
|  | White | 53.1% | 61% |
|  | Multiracial or other | 20.6% | 2% |
|  |  |  |  |
|  | With Children | 21.1% | 23% |
|  |  |  |  |
|  | Same-sex Relationship | 8.9% | 6% |
|  | Other-sex | 91.9% | 94% |
|  |  |  |  |
|  |  |  |  |
| Variables *M(SD)* |  |  |  |
|  | Color-blind Racial Ideology | 48.04(19.26) | 59.05(19.22) |
|  | Ethnic Identity - Affirmation | 22.32(2.93) | 21.35(4.11) |
|  | Ethnic Identity - Exploration | 18.35(5.82) | 18.46(5.64) |
|  | Ethnic Identity - Resolution | 12.03(3.17) | 12.01(2.75) |
|  | Multiculturalism | 4.31(.59) | 3.68(.89) |
|  | Public Stigma | -.01(.62) | .01(.64) |
|  | Family Stigma | .02(.60) | -.05(.55) |
|  | Friend Stigma | -.03(.54) | .07(.66) |
|  | Relationship Quality | 6.12(.83) | 6.01(1.01) |

**Table 2**

*Fit Indices for 1-6 Class Models*

|  | Parsimony Criteria | | | | Clustering Criteria | | | |
| --- | --- | --- | --- | --- | --- | --- | --- | --- |
| Classes | AIC | BIC | CAIC | ssBIC | CLC | NEC | E | ICL.BIC |
| 1 | 8711.46 | 8748.92 | 8758.92 | 8717.20 | 8691.46 | 1.00 | N/A | 8748.92 |
| 2 | 8404.86 | 8468.55 | 8485.55 | 8414.63 | 8423.52 | 0.16 | 0.88 | 8521.20 |
| 3 | 8245.93 | 8335.84 | 8359.84 | 8259.72 | 8298.32 | 0.20 | 0.85 | 8436.23 |
| 4 | 8195.60 | 8311.73 | 8342.73 | 8213.41 | 8241.46 | 0.19 | 0.88 | 8419.60 |
| 5 | 8076.77 | 8219.13 | 8257.13 | 8098.60 | 8148.45 | 0.21 | 0.85 | 8366.81 |
| 6 | 7973.71 | 8142.29 | 8187.29 | 7999.57 | 7975.77 | 0.11 | 0.92 | 8234.35 |
